# Comparative Evaluation of Solubility, Cytotoxicity and Photostability Studies of Resveratrol and Oxyresveratrol Loaded Nanosponges

**DOI:** 10.3390/pharmaceutics11100545

**Published:** 2019-10-20

**Authors:** Nilesh Kumar Dhakar, Adrián Matencio, Fabrizio Caldera, Monica Argenziano, Roberta Cavalli, Chiara Dianzani, Marco Zanetti, José Manuel López-Nicolás, Francesco Trotta

**Affiliations:** 1 Department of Chemistry, University of Torino, via P. Giuria 7, 10125 Torino, Italy; fabrizio.caldera@unito.it (F.C.); marco.zanetti@unito.it (M.Z.); francesco.trotta@unito.it (F.T.); 2 Department of Biochemistry and Molecular Biology-A, Faculty of Biology, 13 University of Murcia - Regional Campus of International Excellence “Campus Mare 14 Nostrum”, E-30100 Murcia, Spain; adrian.matencio@um.es (A.M.); josemln@um.es (J.M.L.-N.); 3 Department of Drug Science and Technology, University of Torino, via P. Giuria 9, 10125 Torino, Italy; monica.argenziano@unito.it (M.A.); roberta.cavalli@unito.it (R.C.); chiara.dianzani@unito.it (C.D.)

**Keywords:** resveratrol, oxyresveratrol, β-cyclodextrin, nanosponges, solubility enhancement, drug-delivery, cytotoxicity, stability studies

## Abstract

Resveratrol and oxyresveratrol are natural polyphenolic stilbenes with several important pharmacological activities. However, low solubility and aqueous instability are the major limitations in their drug delivery applications. In the present work, we demonstrated the encapsulation of resveratrol and oxyresveratrol with nanosponge to improve solubility and stability. Several characterization techniques were used to confirm the encapsulation of both drug molecules within the nanosponges. The high encapsulation efficiency of resveratrol (77.73%) and oxyresveratrol (80.33%) was achieved within the nanosponges. Transmission electron microscopy suggested uniform spherical size particles of resveratrol and oxyresveratrol loaded nanosponges. Compared to free drugs, better protection against UV degradation was observed for resveratrol-loaded nanosponge (2-fold) and oxyresveratrol-loaded nanosponge (3-fold). Moreover, a higher solubilization of resveratrol- and oxyresveratrol-loaded nanosponges lead to a better antioxidant activity compared to drug molecules alone. Cytotoxicity studies against DU-145 prostate cancer cell lines further suggested improved activity of both resveratrol and oxyresveratrol-loaded nanosponges without any significant toxicity of blank nanosponges.

## 1. Introduction

**Figure 1:**
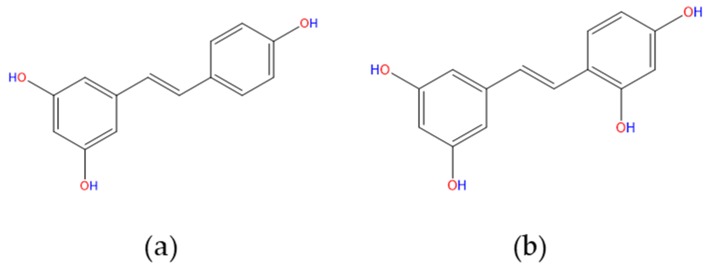
Structure of (**a**) resveratrol and (**b**) oxyresveratrol.

Both resveratrol (*trans*-3,4′,5-trihydroxystilbene; RES, Figure 1a) and oxyresveratrol (*trans*-2′,3,4′,5-tetrahydroxystilbene, OXY, Figure 1b) are naturally occurring polyphenolic stilbenes obtained from a variety of plant sources [1]. RES was first isolated from *Veratrum grandiflora* O. loes [2] and later, has been discovered in several other plants such as peanuts, mulberries, and, *Vitis vinifera* (grape juice) in high concentrations [3]. Moreover, OXY was isolated naturally from mulberry (*Morus Alba L*.) and *Artocarpus lakoocha* Roxburgh (Moraceae) in abundance [4,5].

RES and OXY exhibit antioxidant, anti-inflammatory, neuroprotective and, anticancer activity [6,7]. Furthermore, RES demonstrates cardiovascular benefits, antibacterial and antifungal activity as well [8]. Similarly, other benefits of OXY are hepatoprotection, tyrosinase inhibition, and antiviral activity against herpes simplex virus [9,10,11]. However, both drugs exhibit several challenges in drug delivery as RES and OXY are chemically unstable molecules because of their photosensitivity and undergo oxidative degradation in aqueous solution. Moreover, RES has low aqueous solubility and OXY shows low bioavailability which further limit their therapeutic applications [12,13]. Thus, there is a need to overcome these challenges related to RES and OXY for drug applications.

Several researchers have demonstrated the ability of cyclodextrin to form an inclusion complex with a variety of hydrophobic drug molecules. The encapsulation of RES and OXY with natural and modified cyclodextrin is also reported earlier [14,15]. Duarte et al., demonstrated the preparation of the inclusion complex of RES with methyl-β-cyclodextrin to improve its solubilization [16]. In another study, the effect of β-cyclodextrin and 2-hydroxypropyl-β-cyclodextrin on solubility and stability of OXY was studied [17]. However, nephrotoxicity of natural cyclodextrin, inability to accommodate macromolecules and limitation in providing a slow drug release profile requires an alternative delivery system [18].

Native cyclodextrin crosslinked with several crosslinkers such as diphenyl carbonate (DPC), pyromellitic dianhydride (PMDA), hexamethylene diisocyanate, and 1,1′-carbonyldiimidazole (CDI) leads to the formation of porous, three-dimensional, hypercrosslinked polymers called cyclodextrin nanosponges (CDNSs). The presence of internal cavities of cyclodextrin and interstitial cavities formed by crosslinking of several cyclodextrin molecules with crosslinker help to accommodate a large number of guest molecules compared to native cyclodextrins. The encapsulation of acetyl salicylic acid with native cyclodextrin and NSs was studied earlier and suggested that high entrapment efficiency was observed with NSs compared to β-cyclodextrin [19].

The properties of nanosponge can be tailored by changing the concentration and type of crosslinker which helps to provide a slow and consistent drug release profile that cannot be achieved by native cyclodextrins. The encapsulation of drug molecules within the CDNSs alter aqueous solubility, permeability, release profile, and prevent chemical degradation due to protection from the outer environment [20,21]. Argenziano et al. showed that the encapsulation of imiquimod with CDNSs provides slower drug release compared to carboxymethyl-β-cyclodextrin, and 2-hydroxypropyl-β-cyclodextrin [22].

CDNSs demonstrated the capabilities of encapsulating hydrophobic and hydrophilic drug molecules. Carbonate-based NSs prepared from different crosslinkers have been utilized earlier to improve solubilization, drug release and stability of curcumin [23], nifedipine [24], chrysin [25], and quercetin [26]. The effect of CDI based carbonate nanosponge on the permeability of resveratrol and its application in buccal and topical drug delivery has been evaluated earlier [27].

In the present study, the preparation of carbonate nanosponges of cyclodextrin has been carried out and its effect on the solubility, release profile, photostability, antioxidant and cytotoxicity activity of RES and OXY has been evaluated.

## 2. Materials and Methods

The β-cyclodextrin (β-CD) was a kind gift from Roquette Italia (Cassano Spinola, Italy). Resveratrol and 1,1′-carbonyldiimidazole (CDI) were purchased from Sigma-Aldrich (Milan, Italy). Oxyresveratrol was purchased from TCI Europe. DU-145 prostate cancer cell lines were purchased from ATCC (Manassas, VA, USA) and Cell culture reagents were purchased from Gibco/Invitrogen (Life Technologies, Paisley, UK). Unless otherwise specified, all other chemicals and reagents used were of analytical grade.

### 2.1. Synthesis of β-Cyclodextrin Nanosponge

Briefly, β-CD (5 g) was dissolved in *N*,*N*-dimethylformamide (30 mL) and CDI (2.852 g) were added into it (1:4 molar ratio). The reaction was performed at 90 °C for 3 h to obtained solid monolithic mass which was crushed and washed with water followed by acetone to remove the unreacted monomers [28]. Furthermore, nanosponges were purified in ethanol by Soxhlet extraction for 24 h, air-dried and utilized for further studies.

### 2.2. Solubilization of RES and OXY

The solubilization of RES and OXY was studied in the presence of nanosponge. An excess quantity of RES and OXY was suspended in water (5 mL) and a fixed quantity of nanosponge was added into it. Samples were stirred at room temperature for 24 h in dark and later, the supernatant was collected after centrifugation at 6000 rpm for 20 min, filtered and analyzed on HPLC to determine RES and OXY concentration as stated in Section 2.4.

### 2.3. Preparation of RES- and OXY-Loaded Nanosponge

An aqueous suspension of nanosponges was prepared (10 mg/mL) and required quantity of RES or OXY was added into it at different weight ratios of 1:2, 1:4, and, 1:6 (*w*/*w*; drug: nanosponge). Samples were sonicated for 15–20 min and kept for stirring in dark at room temperature for 24 h. The supernatant was collected after mild centrifugation and dialyzed for few minutes to remove the uncomplexed drug. It was further freeze-dried to collect solid powder which was subjected to characterization.

### 2.4. Quantitative Determination of RES and OXY by HPLC

The quantitative determination of RES and OXY was carried out by an HPLC system (PerkinElmer, Waltham, MA, USA) equipped with a UV detector (Flexar UV/Vis LC spectrophotometer). A reversed-phase phenomenex C18 analytical column (4.6 mm × 250 mm, 5 µm) was used for both drugs. Two different mobile phases were used for the quantification of RES and OXY. The mobile of RES consisted of a mixture of 0.5% acetic acid in methanol and water (52:48, *v*/*v*). A mixture of acetonitrile and 0.5% aqueous acetic acid (27:73, *v*/*v*) was used for OXY [29,30]. The isocratic elution was performed at room temperature, with flow rate and injection volume of 1 mL/min and 20 µL, respectively. The UV detector was set at 305 nm for RES and 326 nm for OXY. The calibration curve was recorded between peak area and concentration of RES and OXY. A linear relation was observed with a correlation coefficient of 1 over a concentration range of 2–10 µg/mL for OXY and a correlation coefficient of 0.9997 over a concentration range of 0.5–2.5 µg/mL for RES was observed.

### 2.5. Determination of RES and OXY Loading Efficiency

The RES loaded NS or OXY loaded NS were taken into a vial containing 1 mL of ethanol and sonicated for 1 h. Later, it was filtered and analyzed on HPLC for RES or OXY content after suitable dilution with the respective mobile phase.

### 2.6. Determination of Particle Size, Polydispersity Index, and Zeta Potential

Dynamic light scattering (Malvern Zetasizer Nano, Worcestershire, UK) was used to determine the mean particle size and polydispersity index at a fixed scattering angle of 90°. All the samples were analyzed at room temperature after suitable dilution with milli-Q water. The same instrument was used to determine the zeta potential of all the samples with the help of an additional electrode placed inside the zetasizer. All the measurements were performed in triplicate.

### 2.7. Differential Scanning Calorimetry

Thermal properties of all the samples were determined using a TA instruments Q200 DSC (New Castle, DE, USA). The standard aluminum pan was used as a reference and samples pan. A fixed quantity of different samples (2–3 mg) was placed in the pan, crimped properly and scanned from 30 to 300 °C at the scanning rate of 10 °C/min under a nitrogen flow of 50 mL/min.

### 2.8. Fourier Transform Infrared Spectroscopy

ATR-FTIR spectra of drugs alone, blank NS and drug-loaded NSs were recorded on PerkinElmer 100 FTIR. Data acquisition was carried out in between 4000–650 cm^−1^ at a resolution of 4 cm^−1^ and collected data were analyzed by spectrum software (PerkinElmer, Waltham, MA, USA).

### 2.9. X-Ray Powder Diffraction Studies

Drugs alone, blank NS and drug-loaded NSs were subjected to diffraction studies using an X-ray diffractometer (Malvern Panalytical X’Pert diffractometer, Worcestershire, UK) using Cu Kα1 as a radiation source. Data were acquired from 3 to 45° (diffraction angle) with a step size of 0.016°. The values of diffraction angle were represented as 2θ.

### 2.10. Determination of Morphology

The morphology of RES and OXY loaded NS was determined by transmission electron microscopy (TEM). A JEOL JEM 3010 (300 KV) transmission electron microscope (JEOL, MA, USA) was used for analysis and the sample was placed on a copper grid. The excess of water was removed by evaporation at room temperature and later subjected to the analysis.

### 2.11. In Vitro Drug Release Profile

The in vitro release profile of OXY and RES was performed using a dialysis bag technique prepared by cellulose membrane (cut-off = 12,400 Da). RES or OXY loaded NSs were dispersed into 2 mL of phosphate buffer pH 7.4 and filled into the dialysis bag. The bag was immersed into 30 mL of phosphate buffer pH 7.4 at 37 ± 0.5 °C with a constant rotation speed of 50 rpm during the experiment. At predetermined time intervals, 5 mL of aliquots were withdrawn. The quantities of RES or OXY present in the aliquots were determined by the HPLC as described earlier. Data were represented as % cumulative drug release vs. time.

The release profile of RES and OXY loaded NS was fitted with different release kinetic models to determine the correlation coefficient. The zero-order release kinetic model was plotted between % cumulative drug release vs. time, first-order release kinetic model was plotted between log cumulative % of drug remaining vs. time, Higuchi–Connors model was plotted between cumulative % drug release vs. square root of time and Korsmeyer–Peppas model was plotted between log cumulative % drug release vs. log time.

### 2.12. Photodegradation Study

The photodegradation of RES and OXY was carried out under a UV lamp. The samples were irradiated under UV light (CAMAG UV LAMP 4; wavelength 320–400 nm, (Alfatech, Genova, Italy) from a fix distance of 10 cm. All the samples were analyzed on HPLC (PerkinElmer, Waltham, MA, USA) at different time intervals according to the HPLC method specified earlier for OXY and RES. A ratio of change in drug concentration (C) against initial drug concentration (C_0_) was determined.

### 2.13. Antioxidant Activity of RES and OXY

Antioxidant activity of OXY, RES, OXY loaded NS and RES loaded NS was determined by DPPH (2,2-diphenyl-1-picrylhydrazyl) inhibition assay. Different concentrations (10–100 µM) of drug alone (RES or OXY) in ethanol and drug-loaded NS in water were prepared. An ethanolic solution of DPPH (0.004%, *w*/*v*) was prepared and 1 mL of DPPH solution was mixed with 1 mL of drug alone or drug-loaded NSs which was incubated for 30 min. All the samples were analyzed by UV–visible spectrophotometer (PerkinElmer Lambda 25, Waltham, MA, USA) at 525 nm. Ethanol (1 mL) was used as a control (without drug). The percentage of DPPH scavenging activity was calculated using the following Equation (1).

% Inhibition = Control absorbance value − Sample absorbance value/Control absorbance value × 100(1)

### 2.14. Cell Viability Studies

DU-145 cell lines were grown as a monolayer culture in RPMI 1640 medium supplemented with 10% fetal bovine serum, 2 mmol/L l-glutamine and 100 U/mL penicillin-streptomycin at 37 °C in a 5% CO_2_ humidified atmosphere.

The percentage inhibition of cell viability by RES and OXY loaded NS was evaluated using 3-(4,5-dimethylthiazol-2-yl)-2,5-diphenyltetrazolium bromide (MTT) assay against DU-145 prostate cancer cells. DU-145 cells (2 × 10^3^/well) were seeded into a 96-well plate and incubated for 24 h at 37 °C in a 5% CO_2_ humidified atmosphere. The cells were treated with increasing concentrations of OXY and RES loaded NS (10–100 µM) for 96 h. The Blank NS was also dispersed in RPMI 1640 medium and treated with the cells as mentioned above. After 96 h, % inhibition of cell viability was determined by recording the absorbance at 570 nm using a microplate reader. The control (i.e., cells that have received no drugs) were normalized to 100%, and the readings from treated cells were expressed as % of viability inhibition. Eight replicates were used to determine each data point and five experiments were performed.

### 2.15. Statistical Analysis

Data were represented by mean ± standard deviation (SD) for each group. The significant difference between the experimental groups was determined by one-way ANOVA followed by Bonferroni correction (data were normally distributed) using GraphPad Prism 5 software (GraphPad Software, San Diego, CA, USA). A *p*-value < 0.05 was considered as statistically significant which was represented by Asteric denotes (* *p* < 0.05).

## 3. Results and Discussion

**Figure 2:**
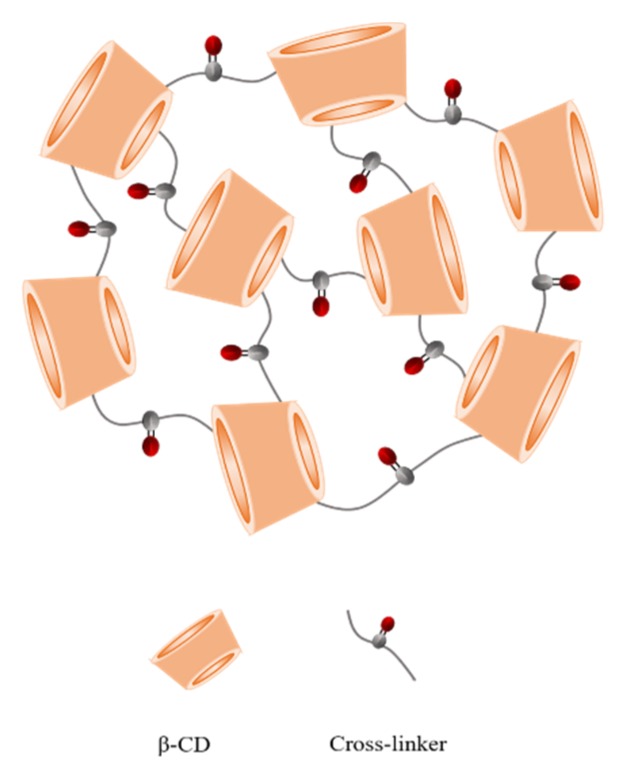
Schematic representation of cyclodextrin nanosponges.

Low aqueous solubility of a drug remains a major challenge in order to develop a drug delivery system. Moreover, a rapid drug release is also associated with dose-related toxicity issues [31,32]. In this work, we demonstrated the enhancement in the solubility, photostability, and cytotoxicity of RES and OXY after encapsulation within the CDNSs. The schematic representation of prepared NSs has been shown in Figure 2.

### 3.1. Characterization of Drug Loaded NSs

**Figure 3:**
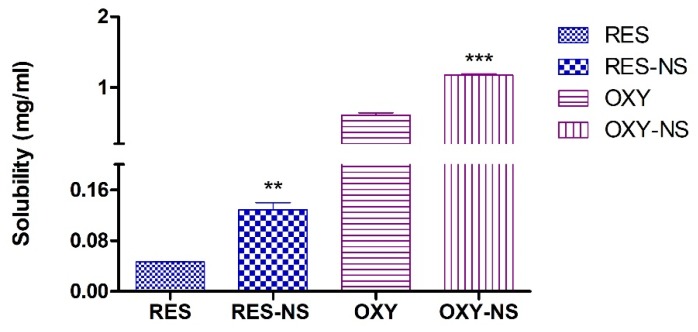
Solubilization of resveratrol (RES) and oxyresveratrol (OXY) in presence of nanosponges (NSs). ** *p* < 0.001, and *** *p* < 0.0001 indicates a significant difference between RES vs. RES-NS or OXY vs. OXY-NS.

The crosslinking density of NSs can be altered by preparing NSs with different crosslinker ratio which subsequently affects the solubilization and drug loading efficiency of the NSs. Recently, we have demonstrated a novel approach to determine the crosslinking density of carbonate nanosponges which suggested that the crosslinking density of CDI nanosponges (1:4) observed was 80% (Supplementary Materials) [33]. The solubilization of RES and OXY alone and in the presence of nanosponge (NS) is shown in Figure 3. The aqueous solubility of RES and OXY was 0.04 mg/mL and 0.6 mg/mL, respectively. Moreover, the encapsulation of RES and OXY with NS leads to enhancement of the aqueous solubility of both RES and OXY. Indeed, RES exhibited poor aqueous solubility and high solubilization of RES was achieved compared to OXY. The enhancement in the aqueous solubility of both RES and OXY could be attributed to the encapsulation in the CD cavities and interstitial spaces of the NSs. The ability of NSs to enhance the solubilization of poorly water-soluble drug molecules has been demonstrated earlier [34,35].

RES and OXY loaded NS were prepared by taking a different weight drug into NS. Weight ratios of 1:2, 1:4, and 1:6 (*w*/*w*) were used. The drug loading of RES was 9.47% at 1:2 *w*/*w*, 13.84% at 1:4 *w*/*w* and 14% at 1:6 *w*/*w*. A similar pattern was exhibited by OXY loaded NS, 11.93% at 1:2 *w*/*w*, 16.06% at 1:4 *w*/*w* and 16.78% at 1:6 *w*/*w*. In both cases, a significant difference (*p* < 0.05) in the drug loading was observed between 1:2 *w*/*w* and 1:4 *w*/*w* for RES and OXY. However, no significant difference of drug loading between 1:4 *w*/*w* and 1:6 *w*/*w* was observed for both RES and OXY, probably it might be because of the achievement of the saturation solubility of RES and OXY. Similar findings, suggesting the effect of change in the weight of NSs with respect to drug molecules and its effect on the % loading of atorvastatin calcium, was reported by Zidan and co-workers [36].

Because of the high drug loading, RES loaded NS (RES-NS) and OXY loaded NS (OXY-NS) at 1:4 *w*/*w* ratio were selected for further studies. The average particle size of RES-NS and OXY-NS was shown in Table 1. The particle size distribution was also observed in the desired range for RES-NS and OXY-NS. The zeta potential of RES-NS and OXY-NS was sufficient enough to make a stable suspension on storage.

**Figure 4:**
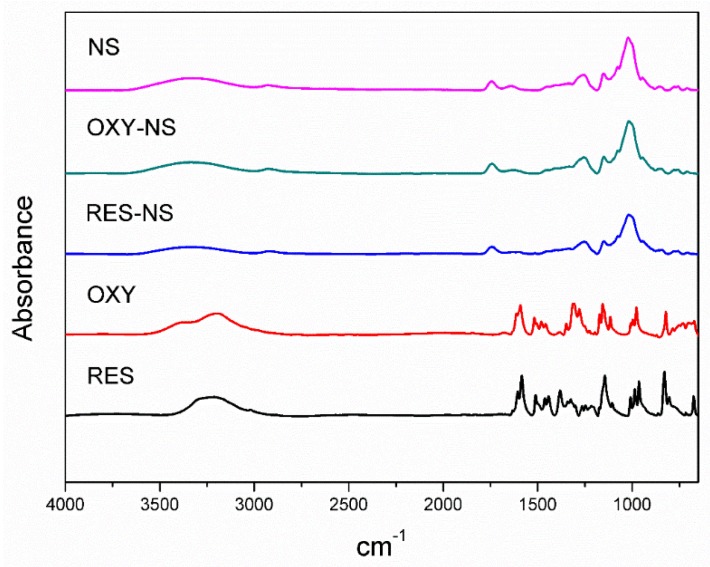
FTIR spectra of RES, OXY, RES-NS, OXY-NS, and blank NS.

The FTIR spectra of drugs alone and drug-loaded NSs were shown in Figure 4. The characteristic peak of NS was observed at 1743 cm^−1^ because of the stretching vibrations of the carbonyl group. RES showed characteristic vibrations at 3209 cm^−1^ (O–H stretching), 3019 cm^−1^ (C–H stretching of phenyl ring), 1605 cm^−1^ (C=C stretching), 1322 cm^−1^ (O–H bending) [37].

Major characteristic peaks of RES were disappeared in case of RES-NS and a significant shift in O-H stretching was observed at 3300 cm^−1^. Because of the structural similarity, OXY exhibited little change in characteristic vibrations at 3192 cm^−1^ (O–H stretching), 3038 cm^−1^ (C–H stretching of phenyl ring), 1611 cm^−1^ (C=C stretching), 1325 cm^−1^ (O–H bending) [17]. However, OXY-NS showed a significant shift in O–H stretching vibrations at 3200 cm^−1^, a shift in the characteristic vibrations of RES-NS and OXY-NS might be due to the encapsulation of drug within the NSs [38].

**Figure 5:**
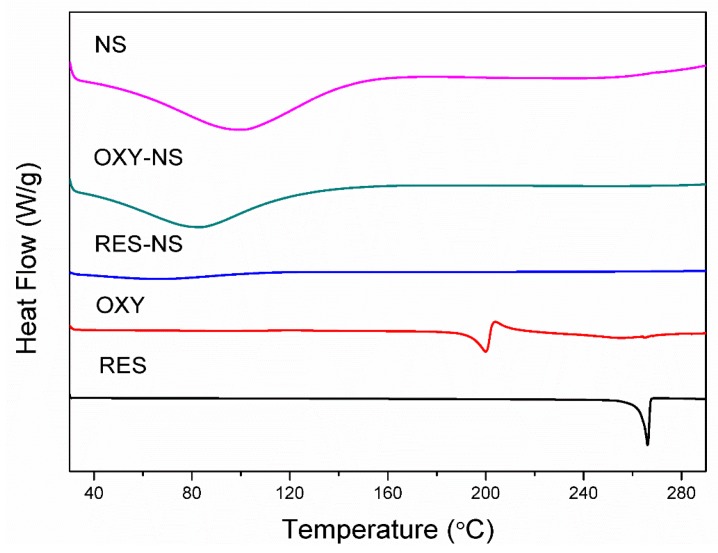
DSC thermograms of RES, OXY, RES-NS, OXY-NS and blank NS.

Blank NS was stable and does not undergo any endothermic transition as shown in Figure 5. RES alone showed an endothermic melting peak at 266.49 °C and OXY exhibited endothermic melting at 203.37 °C [39,40]. However, RES-NS and OXY-NS do not show any endothermic melting transitions because of possible encapsulation of RES and OXY within NSs. This endothermic behavior is in agreement with previously reported findings and suggested that the disappearance of endothermic melting could be attributed to the formation of inclusion complex between drugs and NSs [41]. Moreover, the encapsulation of drugs within NSs leads to the amorphization of drug molecules which was further validated by PXRD.

**Figure 6:**
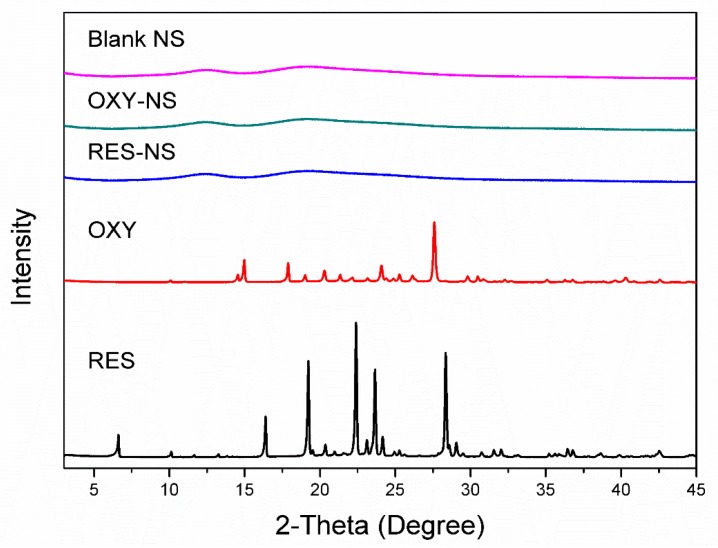
PXRD pattern of RES, OXY, RES-NS, OXY-NS and blank NS.

The physical state of the samples was determined by PXRD studies as shown in Figure 6. Diffraction pattern of RES showed crystalline structure due to the presence of sharp and intense peaks at a 2θ angle of 6.61, 16.38, 19.24, 22.38, 23.10, and 28.33.

Moreover, OXY also showed the crystalline structure and sharp peaks were observed at a 2θ angle of 14.56, 17.87, 20.30, 21.34, 24.09, and 27.60. The PXRD pattern of both drugs is in agreement with the previous findings [40,42] However, the PXRD pattern of NS alone demonstrated its amorphous nature. RES-NS and OXY-NS do not exhibit any intense peaks in their respective PXRD pattern, which confirmed that both drugs were molecularly dispersed in the amorphous state within the NSs [43].

**Figure 7:**
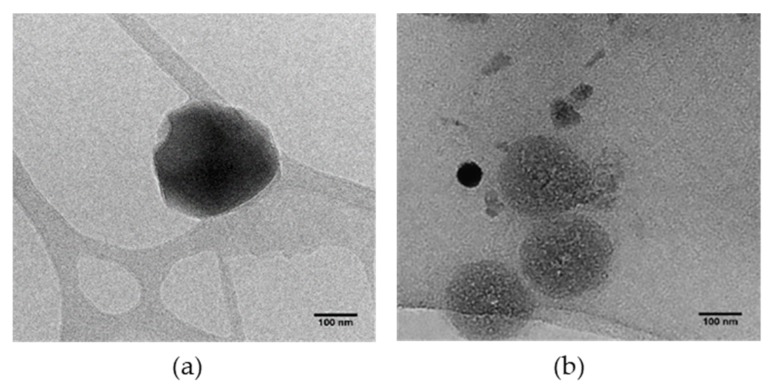
TEM images of (**a**) RES-NS and (**b**) OXY-NS.

Figure 7 represents TEM images of RES-NS and OXY-NS which confirms uniform size spherical particles. The particle size of RES-NS and OXY-NS obtained with TEM is in agreement with the DLS data (200–250 nm).

### 3.2. In Vitro Drug Release Profile

**Figure 8:**
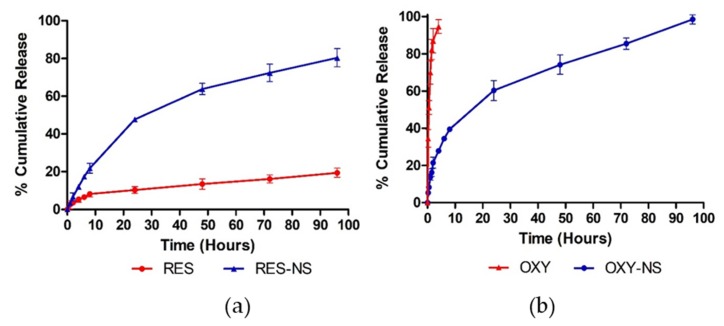
The release profile of (**a**) RES vs. RES-NS and (**b**) OXY vs. OXY-NS.

The in vitro release profile of RES-NS and OXY-NS is shown in the Figure 8. From the dissolution profile of RES-NS, it was evident that RES-NS showed higher release compared to RES alone. RES-NS showed almost five-fold (47.74%) higher drug release compared to RES alone (10.3%) within the first 24 h. The release profile of RES-NS clearly indicated the high solubility of RES that can be attributed to high solubilization and subsequent amorphization after encapsulation within NSs [44]. In contrast, OXY alone showed rapid and uncontrolled drug release in which equilibrium was achieved within a few hours. However, OXY-NS showed a slow and uniform release profile without any initial burst effect. OXY-NS showed 60% of drug release in the first 24 h, the slower release of OXY might be due to encapsulation which further leading to strong complexation within the NSs. Moreover, a slow anticancer drug release without an initial burst effect can decrease dose-related side effects which was achieved with both RES and OXY [45,46].

The release profile of RES-NS and OXY-NS was fitted with different release kinetic models to demonstrate the mechanism of drug release (Table 2). The drug release profile of RES-NS and OXY-NS was best fitted to Higuchi–Connors release kinetics models indicating that the mechanism of drug release was diffusion from the NSs. It was reported earlier as well that the diffusion acts as a driving force for the release of drug molecules from NSs [47].

### 3.3. Photodegradation Study

**Figure 9:**
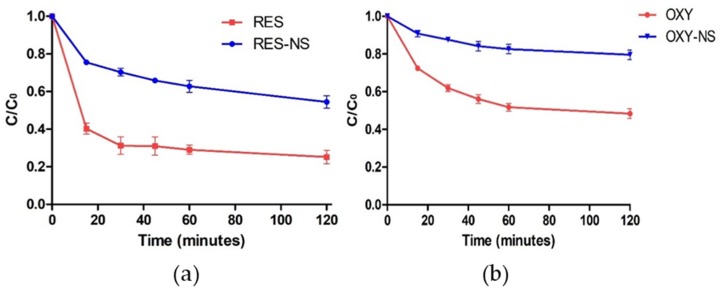
Photodegradation study of (**a**) RES vs. RES-NS and (**b**) OXY vs. OXY-NS.

Photodegradation study of RES and OXY was demonstrated in Figure 9. It has been widely reported that cyclodextrin-based nanocarriers provide protection from UV degradation for the host molecules [48]. Bertacche et al. demonstrated the photostability of RES inclusion complex prepared with natural and modified cyclodextrin [49]. In agreement with these findings, RES and OXY alone showed 59.7% and 27.5% degradation, respectively within 15 min because of the direct exposure to the UV light. Indeed, it was evident that RES-NS and OXY-NS showed better protection from UV light. RES-NS showed two-fold and OXY-NS showed three-fold protection compared to respective alone drugs in solution. The encapsulation of RES and OXY within NSs prevents their exposure to UV light thus leads to protection of both the drug molecules [27,50].

### 3.4. Antioxidant Activity

**Figure 10:**
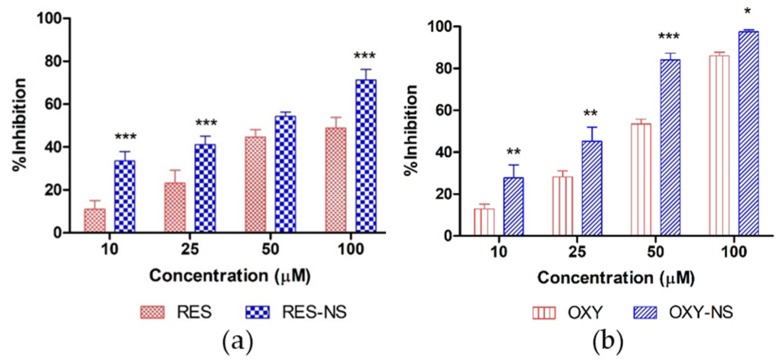
DPPH inhibition activity of (**a**) RES vs. RES-NS and (**b**) OXY vs. OXY-NS. * *p* < 0.05, ** *p* < 0.01, and *** *p* < 0.001 indicates a significant difference between RES vs. RES-NS or OXY vs. OXY-NS at the same concentrations, two-way ANOVA, followed Bonferroni correction.

Antioxidant activity of RES and OXY was determined by their inhibition potential against DPPH. DPPH accepts protons or electrons from the antioxidant and undergoes discoloration which can be measured quantitatively by the change in the absorbance values with respect to control [51]. Figure 10 presents DPPH inhibition of RES, OXY, RES-NS, and OXY-NS. Indeed, OXY alone showed better antioxidant activity compared to RES alone, this might be due to the presence of an additional hydroxyl group which helps to stabilize DPPH free radical [4]. Moreover, encapsulation of RES and OXY further showed significant enhancement in antioxidant activity for both RES-NS and OXY-NS because of the higher solubilization of both drugs which readily provides protons for the reaction with DPPH. Above findings are in agreement with previously reported results which showed that higher DPPH inhibition was observed with quercetin-NS inclusion complex compared to quercetin alone [26].

### 3.5. Cell Viability Study

**Figure 11:**
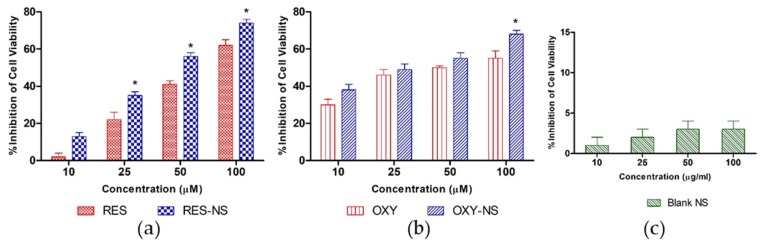
Cell cytotoxicity study of (**a**) RES vs. RES-NS (**b**) OXY vs. OXY-NS, and (**c**) blank NSs after 96 h. * *p* < 0.05 indicates a significant difference between RES vs. RES-NS or OXY vs. OXY-NS at the same concentrations.

Dei-Cas and Ghidoni stated the effect of stilbenes in the treatment of cancer [52]. Moreover, Kuwajerwala et al. demonstrated the antiproliferative effect of RES on prostate cancer and suggested that RES inhibits cell cycle progression to exhibit anticancer effects [53]. To confirm the cytotoxicity of RES-NS and OXY-NS, DU-145 prostate cancer cells were treated with samples for 96 h as shown in Figure 11. All the samples showed an increase in cell toxicity in a concentration-dependent manner. Moreover, it was evident that RES-NS and OXY-NS showed higher toxicity compared to RES and OXY alone. A significant difference (*p* < 0.05) in the cytotoxicity for RES-NS was observed compared to RES alone after 96 h at concentrations of 25, 50 and 100 µM, respectively. Moreover, OXY-NS showed a significant cytotoxicity (*p* < 0.05) compared to OXY alone at the highest concentration.

We have also demonstrated that NSs alone do not produce cytotoxic effects on DU-145 prostate cancer cells after 96 h (Figure 11c). The higher cytotoxicity of RES-NS and OXY-NS attributed to the low particle size of nanocarrier and high solubilization of RES or OXY that lead to a change in the physicochemical properties of drugs might be responsible for the higher toxicity [54].

## 4. Conclusions

We illustrated the synthesis of cyclodextrin nanosponges and the encapsulation of RES and OXY within the NSs was demonstrated. The inclusion complex formation between RES or OXY with NSs leads to higher solubilization compared to drugs used alone. The effect of different weight ratios of drug to nanosponges on drug loading was studied which confirmed that OXY and RES showed higher drug loading in 1:4 *w*/*w* ratio of drug to nanosponge. The inclusion complex formation of RES and OXY was confirmed by FTIR, DSC, and PXRD. The drug release profile of RES and OXY suggested the capability of nanosponge to solubilize drug molecules and extended their drug release in a controlled manner. It was clearly evident that NSs protect both RES and OXY from outer environment to prevent their degradation under UV light. Antioxidant activity of RES and OXY was further enhanced in the presence of NSs. Higher cytotoxicity was observed for both RES-NS and OXY-NS against DU-145 prostate cancer cells, induced by the change in the physicochemical property of OXY and RES. Moreover, nanosponges alone were biocompatible without any significant cytotoxicity. Above findings clearly suggest that nanosponges can be employed as a delivery vehicle for a variety of drug molecules to improve their solubilization, stability and to control their drug release profile.

## Figures and Tables

**Table 1:** Physicochemical properties of RES-NS and OXY-NSs.

Properties	RES-NS	OXY-NS
**Particle Size (nm)**	213.4 ± 2.45	220.3 ± 7.24
**PDI**	0.32 ± 0.02	0.29 ± 0.08
**Zeta Potential (mV)**	23.6 ± 0.25	22.3 ± 0.90
**Encapsulation Efficiency (%)**	77.73%	80.33%

**Table 2:** Release kinetic models for RES-NS and OXY-NSs.

Models	RES-NS	OXY-NS
	R^2^	R^2^
**Zero Order**	0.904	0.9311
**First Order**	0.9596	0.9136
**Higuchi-Connors**	0.9852	0.9828
**Korsmeyer-Peppas Model**	0.6052	0.7076

## Supplementary Materials

The following are available online at https://www.mdpi.com/1999-4923/11/10/545/s1, Figure S1: Calibration Curve of β-CD and carbonate standard, Figure S2: Reference and sample FTIR spectra, Table S1: Crosslinking density determination.
