# Supplementary material for: Comparative Evaluation of Solubility, Cytotoxicity and Photostability Studies of Resveratrol and Oxyresveratrol Loaded Nanosponges
Source: Pharmaceutics. 2019 Oct 20;11(10):545. doi: 10.3390/pharmaceutics11100545 (PMC6836080; doi:10.3390/pharmaceutics11100545)
Supplement: Supplementary file 1 [file pharmaceutics-11-00545-s001.pdf]

pharmaceutics

Supplementary Materials: Comparative Evaluation of Solubility, Cytotoxicity and Photostability Studies of Resveratrol and Oxyresveratrol Loaded Nanosponges

Nilesh Kumar Dhakar, Adrián Matencio, Fabrizio Caldera, Monica Argenziano, Roberta Cavalli, Chiara Dianzani, Marco Zanetti, José Manuel López-Nicolás and Francesco Trotta

1. Calibration Curve

Beta-cyclodextrin (β-CD and a carbonate standard (diphenyl carbonate; DPC) were mixed together in an increasing molar ratio of 1:1 to 1:8. KBr pellets were prepared by keeping a constant weight for each pellet and FTIR spectra were recorded. A calibration curve was plotted in between I_{1774}/I_{2929} peak ratio vs. molar ratio of β-CD to DPC.

I_{1774} corresponds to the C=O stretching vibration of cross-linker and I_{2929} corresponds to the C–H stretching vibration of β-CD.

A linear calibration curve with a regression coefficient of 0.9971 was obtained.

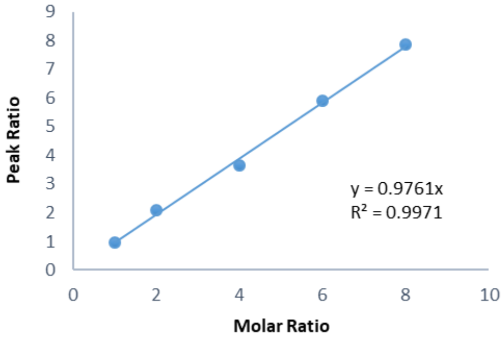

Figure S1. Calibration Curve of β-CD and carbonate standard.

Comparison of reference (β-CD: DPC; 1:4) and sample (nanosponges) spectra are shown below.

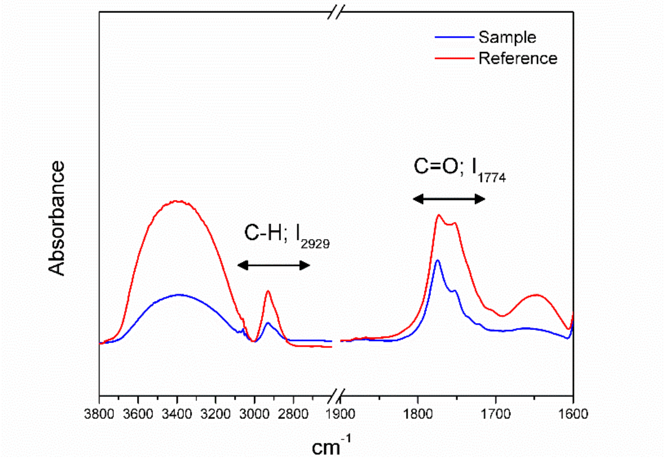

Figure S2. Reference and sample FTIR spectra.

Table S1. Crosslinking density determination.

Nanosponges	Standard		Sample		% Crosslinking = Sample I_{1774}/I_{2929} Value ÷ Reference I_{1774}/I_{2929} Value*100
	Molar Ratio (Crosslinker: β-CD)	Reference I_{1774}/I_{2929} Value	Sample I_{1774}/I_{2929} Value	Calculated Molar Ratio (Crosslinker: β-CD)	
CDNSs	4	3.650	2.920	2.99	80%
